# Artificial intelligence generated clinical score sheets: looking at the two faces of Janus

**DOI:** 10.1186/s42826-024-00206-6

**Published:** 2024-05-16

**Authors:** Cristian Berce

**Affiliations:** Animal Health and Welfare Division, Federal Food Safety and Veterinary Office, Bern, Switzerland

**Keywords:** Score sheets, Preclinical, LLM, Artificial intelligence, In vivo

## Abstract

In vivo experiments are increasingly using clinical score sheets to ensure minimal distress to the animals. A score sheet is a document that includes a list of specific symptoms, behaviours and intervention guidelines, all balanced to for an objective clinical assessment of experimental animals. Artificial Intelligence (AI) technologies are increasingly being applied in the field of preclinical research, not only in analysis but also in documentation processes, reflecting a significant shift towards more technologically advanced research methodologies. The present study explores the application of Large Language Models (LLM) in generating score sheets for an animal welfare assessment in a preclinical research setting. Focusing on a mouse model of inflammatory bowel disease, the study evaluates the performance of three LLM – ChatGPT-4, ChatGPT-3.5, and Google Bard – in creating clinical score sheets based on specified criteria such as weight loss, stool consistency, and visible fecal blood. Key parameters evaluated include the consistency of structure, accuracy in representing severity levels, and appropriateness of intervention thresholds. The findings reveal a duality in LLM-generated score sheets: while some LLM consistently structure their outputs effectively, all models exhibit notable variations in assigning numerical values to symptoms and defining intervention thresholds accurately. This emphasizes the dual nature of AI performance in this field—its potential to create useful foundational drafts and the critical need for professional review to ensure precision and reliability. The results highlight the significance of balancing AI-generated tools with expert oversight in preclinical research.

## Background

Current best practices in animal welfare, particularly in experiments that might cause pain or distress, advocate for the use of clinical score sheets [1]. These sheets are essential for maintaining animal welfare by minimizing distress, and they provide a reproducible, standardized method to evaluate animals, ensuring ethical treatment and scientific integrity.

A score sheet lists specific symptoms and behaviours for monitoring, with intervention guidelines and frequency of animal checks, including model-specific symptoms and intervention thresholds [2]. It quantifies symptoms for objective evaluation, focusing on relevant clinical signs for welfare assessment while avoiding unnecessary details that could cloud interpretation [1]. .

The evolution of Large Language Model(s) (LLM), like the Generative Pre-trained Transformer (GPT) series, has seen significant advancements. GPT-3’s 175 billion-parameter transformer architecture has evolved into GPT-3.5 and GPT-4, showing enhanced accuracy and broader applications in fields like medicine and veterinary science, despite undisclosed parameter counts [3–6].

In this study, I applied validated prompt engineering methods [7] to train LLMs for drafting clinical score sheets, assessing their ability to streamline these animal welfare assessment tools. Prompts, acting as a programming model, enable customization of LLM responses to achieve desired qualitative and quantitative outputs.

## Main text

### LLM prompt design and evaluation

The study and data collection took place between September 29th and December 1st, 2023.

Three LLM “Chat bots” were explored for their potential use to test this hypothesis: Google Bard, a chat based Artificial Intelligence (AI) tool developed by Google LLC (Mountain View, CA, USA) and ChatGTP-4.5 and ChatGPT-4, also chat based AI tools, developed by OpenAI Inc. (San Francisco, CA, USA). These three LLM platforms were selected for their parameter size, development stage, user-friendliness, reliability, and security, their effectiveness being validated in similar data analysis and generation studies [8].

I attempted to generate score sheets for a mouse model of inflammatory bowel disease - ulcerative colitis - through serial identical iterations across the three platforms. I used the DSS model standards [9], completed with inflammation [10] and appearance symptoms [11]. As such the score sheet that I aimed to generate focused on assessing weight loss, stool consistency, and visible fecal blood. Table 1 illustrates the range of symptoms I aimed for the LLM to generate in the clinical score sheet.

**Table 1:** Adaptation of scoring system used by Melgar S. et al.

Score	Body weight (BW) loss (%)	Stool consistency	Visible Fecal Blood
0	No BW loss	Normal	Normal
1	>= 5%	Slightly loose feces	Occasional blood spots
2	>= 10%	Loose feces	Regular blood spots
3	>= 15%	Watery diarrhea	Blood is a consistent component of feces

To quantify the quality of LLM-generated score sheets, I allocated one point (*N* = 1) for each symptom (body weight loss, stool consistency, visible fecal blood) listed in Table 1, with a total of three points (*N* = 3) if all symptoms were included. An additional point (*N* = 1) was given if symptom severity matched model specific symptoms [10], and another (*N* = 1) for the inclusion of intervention guidelines, amounting to a maximum of five points (*N* = 5) per clinical score sheet.

Once a prompt or prompt combination consistently produced similar results, I conducted five (*N* = 5) trials using that prompt per platform in new chats to prevent LLM bias, as LLM chatbots do not remember past conversations.

The study also focused on counting hallucinations in LLM-generated score sheets, defined as instances of inaccuracy or irrelevant content [12, 13]. This measure was crucial for evaluating the LLM’s reliability and its practical use, as hallucinations indicate responses with non-existent, irrelevant, or fabricated information.

After a series of tests I found that the prompt that would yield reproducable results which resemble a real score sheet is an adaptation of the “template pattern” [7].

The “template pattern” that I used included two distinct stages:

In the first stage, I set a frame for the LLM output by describing what a score sheet is and how it should be structured:

“*In future discussions, please remember this explanation and confirm if you understand it without repeating what I wrote: when conducting animal experiments that might cause discomfort or harm, it’s important to use animal health score sheets. These sheets help researchers monitor the animals’ condition based on specific criteria and symptoms. Each symptom is listed with a severity level and a numerical value. Researchers should customize these sheets for each experiment, focusing on how often to check the animals and what symptoms to look for.*

*The first step in making a score sheet is to choose what signs to watch for, like general health indicators and any specific signs related to the experiment. Researchers should track these signs over time for each animal. If the total score from these signs indicates the animal is in pain or discomfort, the researcher must take action, like giving pain relief or rehydration or euthanasia. The duration in which an animal is allowed to have a score consistent with signs of pain or discomfort until the humane endpoint is reached, also needs to be defined. The score sheet should not have irrelevant symptoms listed and scoring needs to be done using a numerical value, making it easy to add up the scores and decide when to intervene*.”

In the second stage, I prompted the LLM to produce a mouse colitis model score sheet based on the described template, specifically requesting a tabular format for clarity:

"*Please generate a colitis mouse model score sheet based on the information I gave above. The score sheet should be in a tabular format*."

### LLM generated clinical score sheet evaluation

After a series of five iterations per LLM, I found that the ChatGPT-4 produced the results with the highest score (21 out of 25 possible points), followed by Google Bard (17 out of 25 possible points) and ChatGPT-3.5 (6 out of 25 possible points). All the iterations are provided in the Supplementary material.

ChatGPT-4 generated score sheets with a consistent structure (Table 2), covering weight loss, stool consistency, and fecal blood, and assigning severity levels with numerical values for a total score to guide interventions. It also included symptoms like abdominal distention and activity level. However, there were significant variations in severity values, intervention thresholds, and humane endpoints, indicating LLM output inconsistency. For instance, in ChatGPT-4 Run 5 (Supplementary material 1), it suggested an unrealistic humane endpoint at a score of > = 10, reflecting severe symptoms not viable in real-life in vivo scenarios due to animal welfare concerns.

**Table 2:** Summary of results from the five runs with ChatGPT-4

ChatGPT-4 (Run Nr.)	Presence of 3 clinical signs (3/3)	Severity of the symptoms (1/1)	Intervention guidelines (1/1)
1	3	1	0
2	3	0	1
3	3	1	1
4	2	1	1
5	2	1	1
Total	13	4	4
Grand Total	21		

ChatGPT-3.5 showed the most deviation from expected results (Table 3) and frequently failed to generate score sheets as instructed (Supplementary material 2). It understood the task, correctly identifying some clinical signs in two of five runs, but produced basic templates lacking specific details. These templates allowed for inputting severity levels and numerical values per experiment needs, prompting users to calculate total scores for action determination, indicating its output was more of a customizable template than a complete score sheet.

**Table 3:** Summary of results from the five runs with ChatGPT-3.5

ChatGPT-3.5 (Run Nr.)	Presence of 3 clinical signs (3/3)	Severity of the symptoms (1/1)	Intervention guidelines (1/1)
1	2	0	0
2	3	1	0
3	0	0	0
4	0	0	0
5	0	0	0
Total	5	1	0
Grand Total	6		

Google Bard’s score sheets outperformed ChatGPT-3.5’s (Table 4) but showed inconsistencies in detail and instruction interpretation for the colitis mouse model. Like ChatGPT-4, it included symptoms like posture and abdominal distention. Although it generally listed symptoms with severity levels and numerical values, there was variation in specificity and value assignment across runs (Supplementary material 3). This inconsistency indicates variability in the model’s comprehension and application of instructions, affecting the score sheets’ comprehensiveness and detail. Google Bard also shared similar issues with ChatGPT-4, such as unrealistic intervention thresholds (see Google Bard – Run 2 in Supplementary material 3).

**Table 4:** – Summary of results from the five runs with Google Bard

Bard (Run Nr.)	Presence of 3 clinical signs (3/3)	Severity of the symptoms (1/1)	Intervention guidelines (1/1)
1	2	1	1
2	1	1	1
3	3	0	0
4	2	1	1
5	2	0	1
Total	10	3	4
Grand Total	17		

Hallucinations in LLM-generated score sheets aligned with their overall performance. ChatGPT-4 showed no hallucinations. ChatGPT-3.5’s inclusion of irrelevant “markdown” or “sql” code in 4 out of 5 runs was classified as hallucinations, with “markdown” in Runs 1 and 2 and “sql” in Runs 3 and 5. Google Bard split the score sheet into multiple tables in 4 out of 5 runs: two tables in Runs 3, 4, and 5, three tables in Run 2, and one table in Run 1. I considered this a partial hallucination, as it still met the basic requirement of a tabular format and as the number of tables required was not specified in the prompt.

### LLM generated clinical score sheet interpretation

LLM development will significantly impact fields like veterinary sciences and preclinical research, particularly in automating tasks like clinical score sheet generation, aligning with the latest AI trends in these areas [4–6]. Creating effective clinical score sheets requires a balance between thorough symptom assessment and practicality [14], which involved guiding LLMs to avoid unnecessary details, a challenge addressed through prompt engineering.

In this study, applying the template pattern was crucial for guiding LLMs to produce structured score sheets, especially because the model doesn’t naturally understand the required format, as discussed by White et al. [7]. This method involved specific instructions for formatting, including sections for symptoms, severity, and interventions. However, as other authors [15] note, this might limit the LLM’s potential to provide additional useful information, highlighting the need for balanced guidance. The study shows LLMs’ efficiency in creating score sheets, maintaining a degree of medical and scientific precision. However, this evaluation was mainly quantitative, focused on a binary assessment of the presence or absence of clinical signs, their severity, and appropriate interventions. This methodology was necessary due to the variability and specificity of clinical score sheets in preclinical research. While I selected a predefined set of clinical symptoms for assessment, it is crucial to acknowledge that these criteria and the corresponding evaluations may need adjustments based on the specific animal model being used [14], emphasizing the importance of professional review and customization of LLM-generated score sheets by experts like laboratory animal veterinarians or animal welfare officers before real-world applications.

The occurrence of hallucinations or the generation of irrelevant or incorrect information remains a challenge in LLM-generated content. This study noted this in the output of ChatGPT-3.5, emphasizing the need for careful review and correction by human experts, as also highlighted by other authors [16]. The score sheets produced by LLM should be seen as a starting point, subject to refinement and validation by experts, rather than as a final product.

This study comparing LLMs like ChatGPT-4, ChatGPT-3.5, and Google Bard highlights the importance of selecting LLMs based on factors such as parameter size and reliability. ChatGPT-4 showed consistent but varied outputs, ChatGPT-3.5 was limited to basic templates, and Google Bard struggled with specificity and clinical sign interpretation. This variation highlights the need for ongoing comparisons as LLMs evolve with reinforcement training techniques [17]. Advances in reinforcement and self-supervised learning have enhanced LLMs’ abilities to autonomously generate complex text, utilizing transformer architecture for better understanding and interaction [18]. A notable limitation of this communication is its focus on the capabilities of LLMs to generate clinical score sheets for only one animal model. Future research could explore how LLMs perform with less common animal models or those with subtler clinical presentations. Additionally, the absence of direct real-world data from LLM-generated score sheets is another limitation. For this study, we relied on indirect real-world data. The choice of this particular model was due to its well-established and characterized clinical scoring. Therefore, we inferred insights from studies using clinical score sheets that mirrored the symptom cluster produced by the LLMs, providing an indirect assessment of their applicability [19–21].

## Conclusions

This study illustrates the potential of Large Language Models (LLM) to generate clinical score sheets in line with the ethical goal of minimizing animal distress during preclinical research. The automation provided by LLM can significantly contribute to the standardization of ethical animal handling practices in a research setting. However, it’s important to emphasize that LLM-generated score sheets should be considered as first drafts or building blocks, rather than final products ready for immediate use. They need to be thoroughly reviewed and adapted by veterinary professionals to ensure accuracy and applicability in specific research contexts. This is particularly important given the observed inconsistencies in LLM results, such as severity levels, intervention thresholds and humane endpoints. Reflecting the duality and transitions symbolized by Janus, this study hints at a growing trend of using AI, specifically LLMs, for tasks like developing clinical score sheets, emphasizing the need for continued research and integration.

## Data Availability

The datasets used and/or analysed during the current study are available from as supplementary materials with the following Digital Object Identifiers: Supplementary material 1: https://doi.org/10.6084/m9.figshare.25326388.v1 ; Supplementary material 2: https://doi.org/10.6084/m9.figshare.25326382.v1 ; Supplementary material 3: https://doi.org/10.6084/m9.figshare.25326385.v1.

## Abbreviations

AI: Artificial Intelligence
GPT: Generative Pre-trained Transformer
LLM: Large Language Model
